# Supplementary material for: ENVIRONMENTS and EOL: identification of Environment Ontology terms in text and the annotation of the Encyclopedia of Life
Source: Bioinformatics. 2015 Jan 24;31(11):1872–4. doi: 10.1093/bioinformatics/btv045 (PMC4443677; doi:10.1093/bioinformatics/btv045)
Supplement: Supplementary Data [file supp_31_11_1872__index.html]

ENVIRONMENTS and EOL: identification of Environment Ontology terms in text and the annotation of the Encyclopedia of Life — ENVIRONMENTS and EOL: identification of Environment Ontology terms in text and the annotation of the Encyclopedia of Life — Supplementary Data 

# ENVIRONMENTS and EOL: identification of Environment Ontology terms in text and the annotation of the Encyclopedia of Life

## Supplementary Data

files

**Files in this Data Supplement:**

- Supplementary Data - pdf file
