# Supplementary material for: ENVIRONMENTS and EOL: identification of Environment Ontology terms in text and the annotation of the Encyclopedia of Life
Source: Bioinformatics. 2015 Jan 24;31(11):1872–4. doi: 10.1093/bioinformatics/btv045 (PMC4443677; doi:10.1093/bioinformatics/btv045)
Supplement: Supplementary Data [file supp_btv045_SupplementaryMaterial_btv045-2015.pdf]

Data and Text Mining

ENVIRONMENTS and EOL: identification of Environment Ontology terms in text and the annotation of the Encyclopedia of Life

Supplementary Material

Evangelos Pafilis*, Sune P Frankild, Julia Schnetzer, Lucia Fanini, Sarah Faulwetter, Christina Pavloudi, Aikaterini Vasileiadou, Patrick Leary, Jennifer Hammock, Katja Schulz, Cynthia Sims Parr, Christos Arvanitidis and Lars Juhl Jensen*

Contact: pafilis@hcmr.gr; lars.juhl.jensen@cpr.ku.dk

S1 SUPPLEMENTARY METHODS

A. Non-Biased Partitioning of the Encyclopedia of Life

The Encyclopedia of Life (EOL) text content has been used in the ENVIRONMENTS and EOL project both for training and for testing. To facilitate this dual aim a non-biased EOL sectioning pattern applied:

- for training purposes text from EOL Taxon Pages with an even identifier was employed
- for testing purposes text from EOL Taxon Pages with an odd identifier was used

This is reflected in manual synonym addition to the ENVIRONMENTS dictionary (Application Note, section 2), in the curated corpus partitioning (Application Note, section 3) and in the ENVIRONMENTS accuracy evaluation (Application Note, section 4).

B. Encyclopedia of Life Annotation: Selected Sections

The following list comprises the types of EOL Taxon Page section employed in the EOL annotation process. More information on the available EOL Taxon Page section types, called “Subjects”, can be found at: http://eol.org/info/98.

http://rs.tdwg.org/ontology/voc/SPMInfoItems#Behaviour
http://rs.tdwg.org/ontology/voc/SPMInfoItems#Biology
http://rs.tdwg.org/ontology/voc/SPMInfoItems#Conservation
http://rs.tdwg.org/ontology/voc/SPMInfoItems#ConservationStatus
http://rs.tdwg.org/ontology/voc/SPMInfoItems#Description
http://rs.tdwg.org/ontology/voc/SPMInfoItems#Dispersal
http://rs.tdwg.org/ontology/voc/SPMInfoItems#Distribution
http://rs.tdwg.org/ontology/voc/SPMInfoItems#Ecology
http://rs.tdwg.org/ontology/voc/SPMInfoItems#GeneralDescription
http://rs.tdwg.org/ontology/voc/SPMInfoItems#Habitat
http://rs.tdwg.org/ontology/voc/SPMInfoItems#LifeCycle
http://rs.tdwg.org/ontology/voc/SPMInfoItems#Migration
http://rs.tdwg.org/ontology/voc/SPMInfoItems#Physiology
http://rs.tdwg.org/ontology/voc/SPMInfoItems#PopulationBiology
http://rs.tdwg.org/ontology/voc/SPMInfoItems#Reproduction
http://rs.tdwg.org/ontology/voc/SPMInfoItems#TaxonBiology
http://rs.tdwg.org/ontology/voc/SPMInfoItems#TrophicStrategy
http://www.eol.org/voc/table_of_contents#Development
http://www.eol.org/voc/table_of_contents#Wikipedia
http://eol.org/schema/eol_info_items.xml#Notes
http://eol.org/schema/eol_info_items.xml#TypeInformation

C. Environment Ontology (ENVO) Resources

The ENVIRONMENTS dictionary is based on the name and synonym information available in the basic version of the Environment Ontology (ENVO).

The latest basic ENVO file in the Open Biological and Biomedical Ontology format (.obo) is available at: http://purl.obolibrary.org/obo/envo/subsets/envo-basic.obo.

A copy of the June 2013 ENVO basic .obo file used in ENVIRONMENTS development can be found at: http://environments.hcmr.gr/envo-basic.2013-06-14.obo

More information on ENVO formats and download possibilities can be found at:
http://environmentontology.org/downloads

S2 SUPPLEMENTARY EVALUATION

A. Mention Detection ENVIRONMENTS Evaluation

The following table presents the mention detection evaluation of the ENVIRONMENTS tagger a. for the complete curated corpus and b. for each curated corpus section type.

Dataset – Page Section	#TP	FP	FN	Precision	Recall	F1:
Complete corpus – All page sections	2776	387	829	87.80%	77.00%	**82.00%**
Migration	34	3	1	91.90%	97.10%	**94.40%**
Habitat	1408	180	371	88.70%	79.10%	**83.60%**
Description	10	3	1	76.90%	90.90%	**83.30%**
Distribution	944	108	322	89.70%	74.60%	**81.40%**
Taxon Biology	129	24	40	84.30%	76.30%	**80.10%**
General Description	136	26	50	84.00%	73.10%	**78.20%**
Dispersal	8	1	4	88.90%	66.70%	**76.20%**
Trophic Strategy	75	20	27	78.90%	73.50%	**76.10%**
Biology	15	6	6	71.40%	71.40%	**71.40%**
Reproduction	14	13	5	51.90%	73.70%	**60.90%**
Ecology	3	3	2	50.00%	60.00%	**54.50%**

B. Definition of Agreement on ENVO Term

As stated in Section 4 (“Performance Evaluation”) of the main manuscript, we evaluated if the matches that were considered true positive for the recognition task also agreed on which ENVO terms to link the match to. To this end we defined agreement to imply that the tagger and the human annotator agreed on at least ENVO term. This implies that when the tagger and the annotator agreed on at least one ENVO term for a given match, additional ENVO terms assigned for that match by only the tagger or the annotator were not counted as disagreements. Consistent with this, a single match only counts as one agreement no matter how many ENVO terms the tagger and the annotator agree on, and a single match for which the tagger and annotator do not agree on any ENVO terms only counts as one disagreement.

We decided on this definition of agreement for two reasons. First, it ensures that all matches in the document count equally, independently of the number of ENVO terms it is linked to. This is desirable as the agreement can then be interpreted as the percentage of matches associated with a correct ENVO term. Second, it avoids counting it as a disagreement, for example, when the tagger links a word such as *grasslands* both to the respective feature (e.g. grassland [ENVO:00000106]) and to the corresponding biome (e.g. grassland biome [ENVO:01000177]), whereas the annotator linked it only to the feature.

S3 Software Documentation

A. Availability

The ENVIRONMENTS tagger software is available under the open-source BSD license and can be found at http://environments.hcmr.gr

B. Dependencies Required for Compiling ENVIRONMENTS

The ENVIRONMENTS software is coded in C++. In addition to the libraries that are part of any C++ installation, it makes use of two extensions: the C++ Technical Report 1 (TR1) library extensions and the BOOST library (http://www.boost.org/).

C. ENVIRONMENTS: Basic Functionality and Output File Format

To invoke ENVIRONMENTS only one parameter is required: the path to a folder containing the plain text (.txt) files to be processed.

```
./environments_tagger documents_directory > matches_file
```

The output of the text file processing is described in **Figure S1**.

D. ENVIRONMENTS: Adjustment and Customization

1. Manual Synonym Addition

A dictionary mapping environment descriptors to ENVO terms lies at the core of the ENVIRONMENTS tagger.

The term name information available in ENVO has been stored in two tab-separated-value text files *environments_entities.tsv*, *environments_names.tsv* .The former is a mapping file of ENVIRONMENTS internal identifiers to ENVO identifiers, while the latter maps the internal identifiers to available names.

ENVIRONMENTS loads the previously mentioned dictionary files on the runtime. Without any recompiling, synonyms found to be missing could be added by manipulating these files. This process could add pieces of information, that may not be available in ENVO.

2. Dictionary Orthographic Expansion, Flexible Match and Stopword List

Based on the term name information retrieved from ENVO extra synonyms were generated capturing the variable ways ENVO terms may be written in text. To this end, orthographic expansion has taken place (e.g. via the addition of the plural and adjective forms of ENVO term names, like coast – coastal and brackish water – brackish waters). To further improve the term identification, flexible matching, e.g. allowing for arbitrary hyphen and/or space insertions within environment descriptive terms is supported.

All the previous mentioned actions improve term matches in text. However, due to *homonymy* (i.e. names having multiple meanings) the risk of False Positive identifications increases. To safeguard against such phenomenon a manually curated *stopword* list, i.e. a list of words to by ignored upon text processing, is in-place (**Figure S2**).

Similarly to the dictionary files the ENVIRONMENTS stopword list is loaded at runtime. Additional stopword list names, can thus be easily added in *environments_global.tsv*.

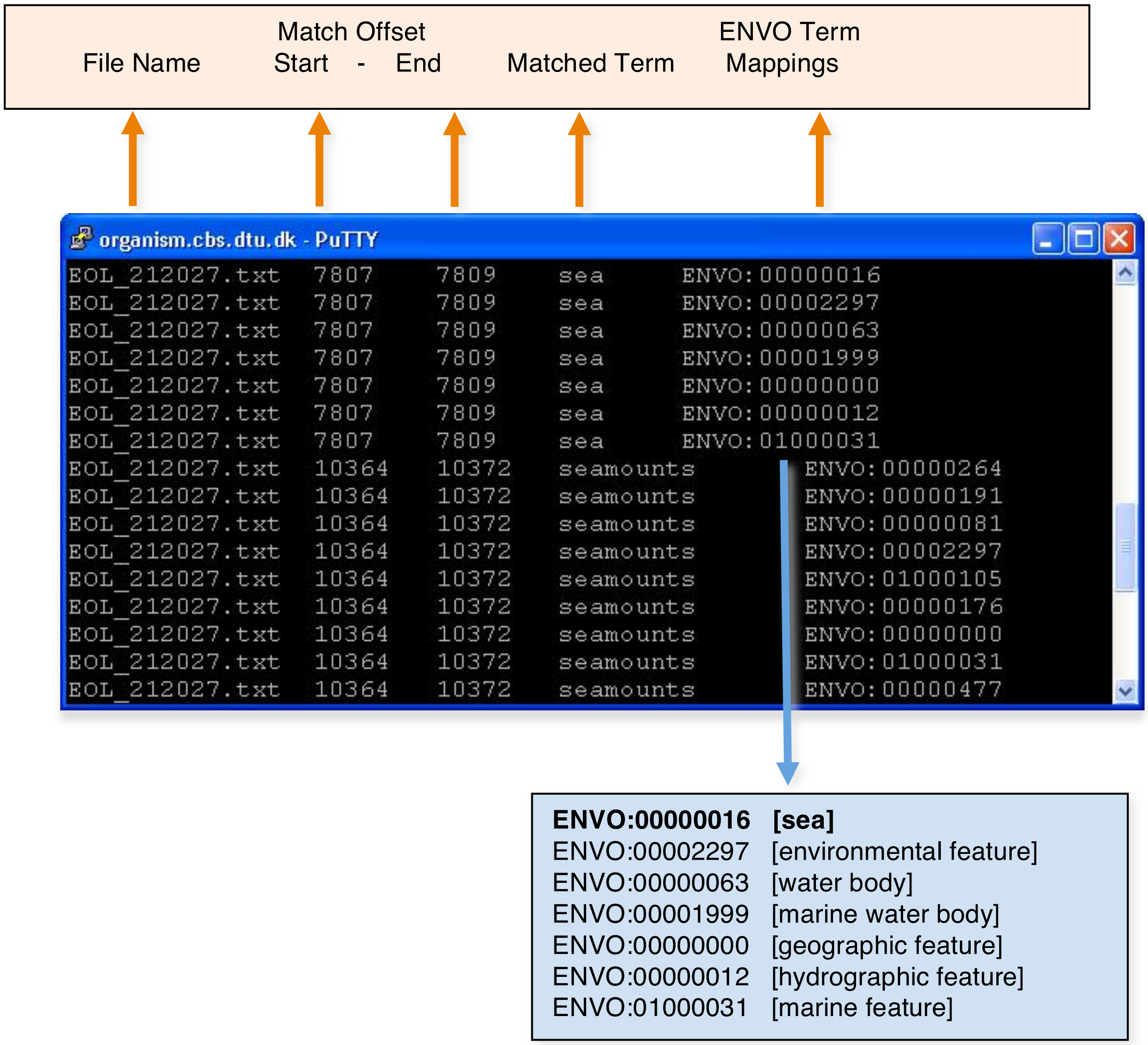

Figure S1: The output of the ENVIRONMENTS tagger is a tab-delimited file comprising 5 columns (terminal window, orange box): the name of the document file, the byte position of the first character of the match, the byte position of the last character of the match, the string matched, and the corresponding ENVO identifier. Each line specifies one match (terminal window); in case of ambiguity, each possible ENVO identifier will be listed as a separate line (with the other columns unchanged). For every matched ENVO term (e.g. blue box in bold) ENVIRONMENTS traverses the ENVO hierarchy and reports all "IS_A" "PART_OF" relationship parents (e.g. blue box non-bold).

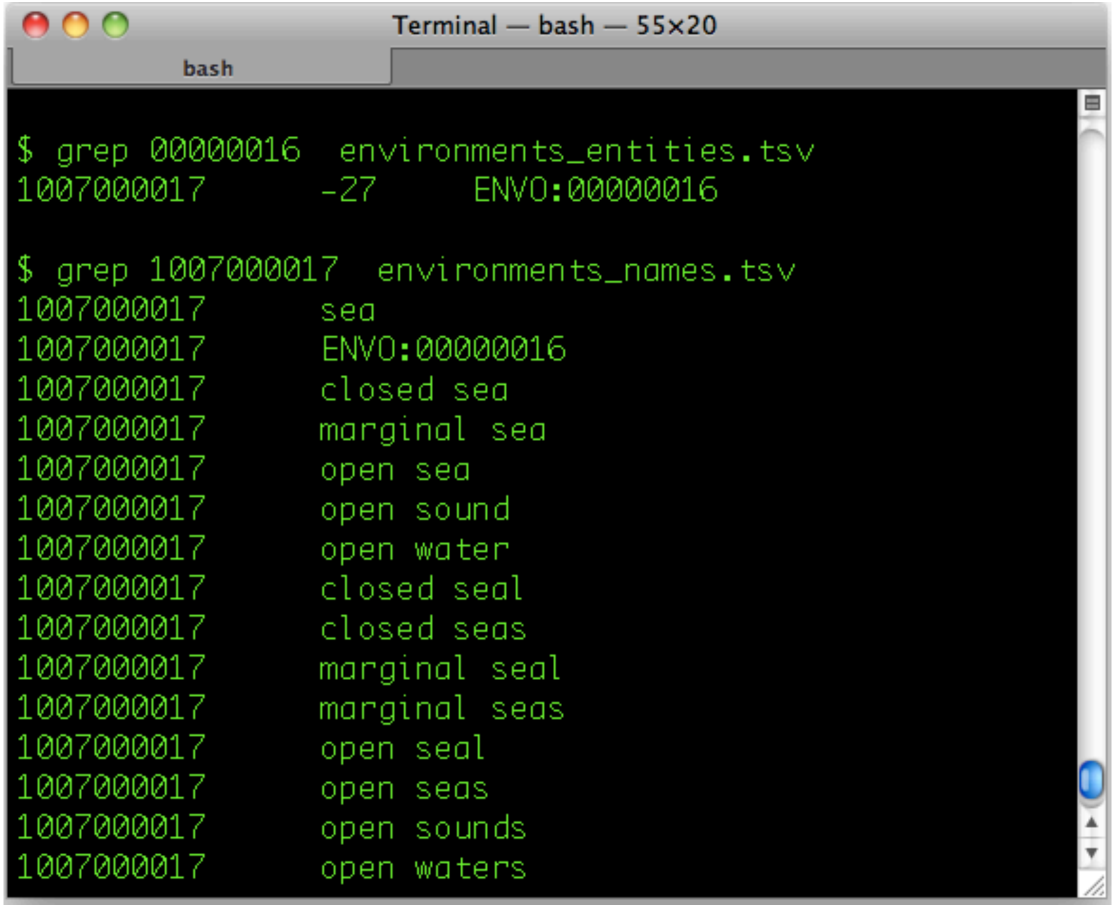

Figure S2: Viewing all the names and synonyms available for an ENVO term («sea», ENVO:00000016 in this examples) is a two step process: a. first the ENVIRONMENTS internal identifier can be retrieved from *environments_entities.tsv*, b. based on this internal identifier all names can then be retrieved from *environments_names.tsv*.

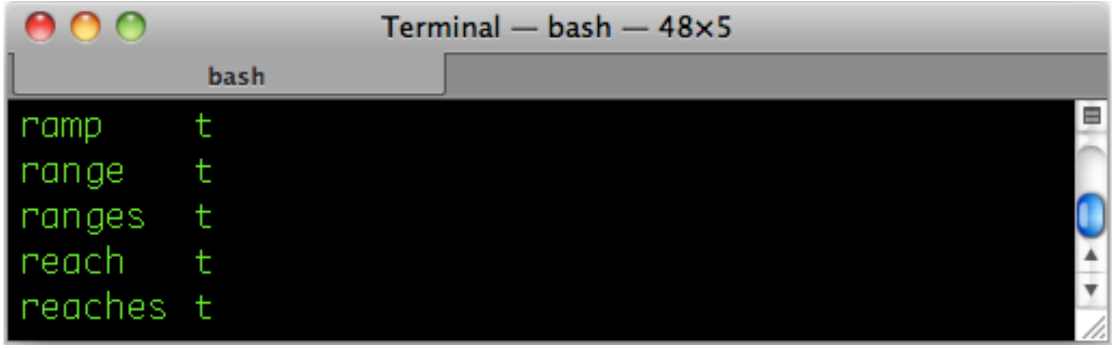

Figure S3: The ENVIRONMENTS stopword *environments_global.tsv* is a plain tab-separated text file. Indicated as “t” (true) are words-to-be-ignored (case-sensitive).
